# Outcomes of catheter ablation of ventricular tachycardia in non-ischemic idiopathic dilated cardiomyopathy: A systematic review and meta-analysis

**DOI:** 10.3389/fcvm.2022.1007392

**Published:** 2022-11-11

**Authors:** Ahmed Ammar, Mohamed Sharief, Khaled Abouelmagd, Omar Riad, Mokhtar Ibrahim

**Affiliations:** ^1^Department of Cardiology, Ain Shams University, Cairo, Egypt; ^2^Department of Cardiology, Worcestershire Acute Hospitals NHS Trust, Worcester, United Kingdom; ^3^Department of Cardiology, Specialized Medical Hospital, Mansoura University, Mansoura, Egypt; ^4^Lancashire Teaching Hospitals NHS Foundation Trust, Preston, United Kingdom; ^5^Department of Cardiology, Dr. Mohammad Alfagih Hospital, Riyadh, Saudi Arabia; ^6^Department of Cardiology, Royal Brompton & Harefield NHS Trust, London, United Kingdom; ^7^University Hospitals of Leicester NHS Trust, Leicester, United Kingdom

**Keywords:** catheter ablation, ventricular tachycardia, non-ischemic dilated cardiomyopathy, meta–analysis, systematic review

## Abstract

**Objective:**

To perform a systematic review and meta-analysis of available trials regarding the outcomes of ventricular tachycardia (VT) ablation in patients with non-ischemic dilated cardiomyopathy (NIDCM).

**Methods:**

A comprehensive database search of large four electronic databases, including PubMed, Cochrane, Scopus, and Institute for Scientific Information network meta-analysis, identified five studies enrolling 666 patients for patients with idiopathic dilated cardiomyopathy (IDCM) underwent catheter ablation (CA) for VT. The short-term outcomes assessed included procedural success, VT non-inducibility and procedural complications, whereas the long-term outcomes assessed included VT recurrence, heart transplantation, antiarrhythmic drugs (AAD) use after ablation and death.

**Results:**

A total of 5 observational studies reported outcomes in 666 patients with NIDCM undergoing VT CA. The complete procedural success was moderately high; 65.5% of the patients (95% CI 0.402- 0.857, *p* < 0.001) and the procedural complications occurred in 5.8% of the patients (95% CI 0.040–0.076, *P* = 0.685). Epicardial mapping and ablation were performed among 61.5% and 37% of patients with NIDCM respectively. During a follow up period of 12 to 45 months, there were VT recurrence in 34.2% of the patients (95% CI 0.301–0.465, *p* < 0.080), death in 20.2% of the patients (95% CI 0.059–0.283, *p* < 0.017) and heart transplantation in 12.9% of the patients (95% CI −0.026–0.245, *P* < 0.012).

**Conclusion:**

Ventricular tachycardia CA is effective and safe approach for management of patients with NIDCM with the epicardial approach to be considered as initial strategy especially in presence of ECG and CMR findings suggestive of epicardial substrate. A multicenter randomized trial is crucial to look at the short- and long-term outcomes of VT ablation in NIDCM especially with the advances in mapping and ablation techniques and predictors of success.

## Highlights

**What is already known on this topic:**
- VT catheter ablation in patients with NIDCM has mixed or even inferior outcomes as compared to those with ICM.
**What this study adds:**
- VT catheter ablation is effective and safe approach for management of patients with NIDCM.
**How this study might affect research, practice, or policy:**
- This meta-analysis highlights the need for multicenter randomized trial to look at the short- and long-term outcomes of VT ablation in patients with NIDCM and predictors of success.

## Introduction

Non-ischemic dilated cardiomyopathy (NIDCM) is commonly associated with ventricular arrhythmias which could be a cause or a result of decompensated heart failure resulting in a significant rise in both morbidity and mortality (1). Guidelines-directed medications and resynchronization devices for heart failure in addition to antiarrhythmic drugs (AAD) use have reduced ventricular tachycardia (VT) significantly in patients with NIDCM (1, 2). Apart from beta-blockers, the AAD including Amiodarone have failed to improve survival, likely because of the long-term side effects counterbalancing the benefits (3, 4).

Catheter ablation has emerged recently as adjunctive treatment to prevent recurrent implantable cardioverter defibrillator (ICD) therapies in patients with NIDCM and VT. The benefit of a successful ablation in preventing VT recurrences and prolonging the survival has been well established (5). However, many observational studies have reported mixed or even inferior outcomes for VT ablation in patients with NIDCM as compared to those with Ischemic Cardiomyopathy (ICM) (6, 7).

Catheter ablation of VT in patients with NIDCM can be challenging because of the complexity of the underlying arrhythmic substrates, with a high prevalence of mid-myocardial and sub-epicardial substrates (8–10). As the number of patients with NIDCM undergoing VT ablation has markedly increased recently (11), it was very important to review the short and long-term outcomes of the VT ablation in patients with NIDCM.

## Methods

This meta-analysis is written in accordance with the Preferred Reporting Items for Systematic Reviews and Meta-Analyses (PRISMA) statement (12) and was registered in PROSPERO (CRD42021235947). The PICOT format (*P* = population, *I* = intervention, *C* = comparator, *O* = outcome, *T* = timing, *S* = setting) was used to derive the key clinical question (13). This question was, “In adults with NIDCM, what are the short- and long-term outcomes of VT catheter ablation?”

### Data source and search strategy

We searched four electronic databases, including PubMed, Cochrane, Scopus and Institute for Scientific Information without language or year restrictions from 1989 until 2020 using the terms “catheter” AND “ablation” AND “ventricular” AND “arrhythmia” OR “dysrhythmia” OR “tachycardia” OR “VT” AND “non-ischemic” OR “non-ischemic” OR “non-ischemic” and “cardiomyopathy” OR “dilated cardiomyopathy” OR “DCM” OR “IDCM.” We scanned the records for duplicates through Endnote x8 and there were 257 duplicated papers.

### Inclusion and exclusion criteria

Two reviewers (AA and MS) independently screened titles and abstracts to select potential full-text articles according to inclusion and exclusion criteria. This review included any original articles that reported outcomes of VT ablation in NIDCM. No restrictions were applied regarding ethnicity, language, country, gender, age, or publication year. Exclusion criteria included papers that reported ischemic cardiomyopathy and non-ischemic cardiomyopathic with other underlying etiologies as HCM, ARVC, and cardiac sarcoidosis. We also excluded commentaries, meta-analysis and review articles, thesis, conferences and books, chapters and papers with unreliable data or duplicated populations. Any disagreement on inclusion was resolved through discussion and arbitrated in conjunction with the senior authors.

Outcomes of interest included short- and long-term outcomes. The short-term outcomes assessed included procedural success, VT non-inducibility with Programmed Electric Stimulation (PES) and procedural complications, whereas the long-term outcomes included VT recurrence, heart transplantation, AAD use after ablation and death.

### Quality assessment

All included studies were assessed by at least two independent reviewers using the quality assessment tool for observational cohort and cross-sectional studies (NIH tool). The reviewers then agreed on a quality rating among; Good, Fair, or Poor, as the guidelines suggest (14).

### Data extraction

Data sheet was built to extract the relevant data, which contained four major sections. The first section included papers’ data and baseline characteristics. The second section contained procedural data and their short-term outcomes. The third section was to identify procedural complications and in-hospital mortality. Finally, the fourth section aimed at pointing out the long-term outcomes of procedures.

Data extraction was performed by two independent reviewers, and the discrepancies between the reviewers were resolved by consensus.

### Statistical analysis

A meta-analysis of proportions of the available main variables was conducted and a Freeman-Tukey’s transformation was applied to establish the variance of raw proportions (15). To incorporate heterogeneity among the included studies, transformed proportions were combined using DerSimonian-Laird random effects model (16). Then the pooled estimates were back-transformed, estimated ER and 95% CI were calculated for each outcome. The heterogeneity of the included studies was evaluated using the Cochran Q and I2 test. Weighted means were calculated by determining the total number of events in each study divided by the total sample size.

## Results

### Search results

**FIGURE 1:**
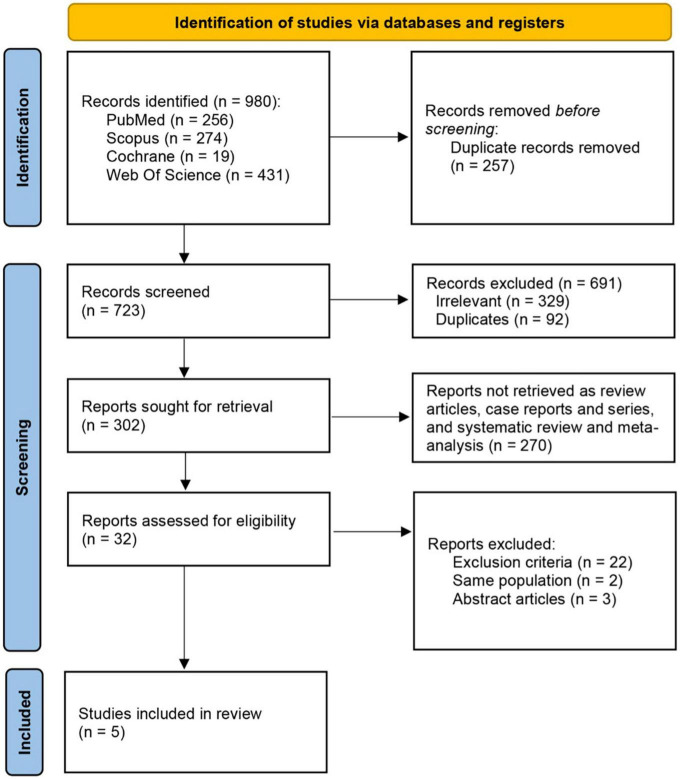
Flow chart of studies identified, screened, and included in the analysis.

A total number of 980 records were retrieved from searching the electronic databases. After the removal of duplicates, 723 studies underwent title and abstract screening. Full text screening of 32 studies revealed five included studies. Figure 1 outlines the search strategy and pathway to the final included studies and baseline study characteristics are provided in Table 1.

**TABLE 1:** Baseline study characteristics.

References	Number	Design	Key inclusion	Acute procedural success definition
Vaseghi et al. (17)	518	Retrospective observational study	Patients with NIDCM who underwent VT ablation (subgroup)	Non-inducibility of any sustained monomorphic VT
Muser et al. (18)	71	Retrospective observational study	Patients with NIDCM and drug refractory electrical storm referred for CA (2005 to 2014)	Non-inducibility of the clinical VT and of any inducible VT apart from non-clinical VTs with a CL < 250 ms
Goya et al. (20)	19	Retrospective observational study	Patients with NIDCM and clinically documented sustained monomorphic VT (subgroup)	Non-inducibility of the clinical VT or all hemodynamically stable VTs in VT mapping group, elimination of the targeted potentials in the substrate mapping group and completion of the designed lines in case of linear ablation
Piers et al. (10)	45	Retrospective observational study	Patients with NIDCM who underwent VT ablation	Non-inducibility of any monomorphic VT
Arya et al. (21)	13	Retrospective observational study	Patients with NIDCM and electrical storm due to monomorphic VT with ICD *in situ* who underwent CA (subgroup)	Non-inducibility of any VT (clinical or non-clinical)

### Quality assessment and publication bias

The overall rating of quality assessment for the included observational studies was good, the median score was 11 with a minimal score eight for one study (Briceño et al) and a maximum score of twelve for four studies (Okubo et al., Vaseghi et al., Kumar et al., and Dinov et al).

### Characteristics of included studies and procedural data

Five Observational studies were included in our review with a total sample size of 666 patients (17–21). All studies were retrospective observational studies. Individual study sample sizes ranged from 13 patients to 518 patients and the mean patient’s age ranged from 56 to 60 years with 93.2% of the studied population of male gender. Table 2 outlines the cohort baseline clinical characteristics.

**TABLE 2:** Baseline clinical characteristics.

References	Total number	Age (y) mean ± SD	Male	ICD/ CRT-D	BB	Amiodarone	Other AAD	LVEF (%) mean ± SD	HTN	DM	HF (NYHA III/IV)	Electrical storm
Vaseghi et al. (17)	518	60 ± 13	500	NR	NR	NR	NR	33 ± 13	44	16	34	37
Muser et al. (18)	71	60 ± 15	62	71	61	53	NR	32 ± 14	31	10	33	71
Goya et al. (20)	19	60.2 ± 15.5	16	19	16	13	10	33.8 ± 10.2	4	2	NR	8
Piers et al. (10)	45	60 ± 16	34	30	33	22	26	44 ± 14	16	5	15	8
Arya et al. (21)	13	56.8 ± 17.8	9	13	13	13	NR	33.1 ± 8.6	NR	NR	NR	13

ICD/CRT-D, implantable cardioverter defibrillator/Cardiac resynchronization therapy pacemaker device; BB, beta blockers; AAD, anti-arrhythmic drugs; LVEF, left ventricular ejection fraction; HTN, hypertension; DM, diabetes mellitus; NR, not reported.

Substrate mapping and Activation mapping were done using the CARTO and Ensite mapping system. Epicardial mapping was reported only in 4 studies and performed in 61.5% of the study population while epicardial ablation was needed in 37% of the patients.

## Outcomes of ventricular tachycardia ablation

### Acute outcomes

**FIGURE 2:**
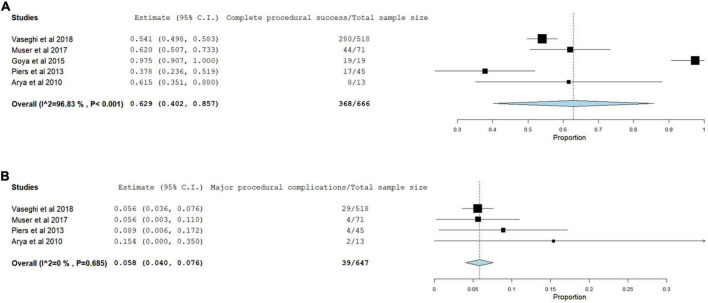
Forest plots for complete procedural success **(A)** and procedural complications **(B)**.

During post-procedural follow-up, complete procedural success defined as complete VT non-inducibility with PES was achieved in 65.5% of the patients (95% CI 0.402- 0.857, *p* < 0.001) (Figure 2A). Whereas the incomplete procedural success and/or failure occurred in 29.7% of the patients. The procedural complications was reported in only 4 studies and occurred in 39 of 647 (5.8%) of the patients (95% CI 0.040–0.076, *P* = 0.685) with the most common is pericardial effusion/cardiac tamponade (Figure 2B).

### long-term outcomes

**FIGURE 3:**
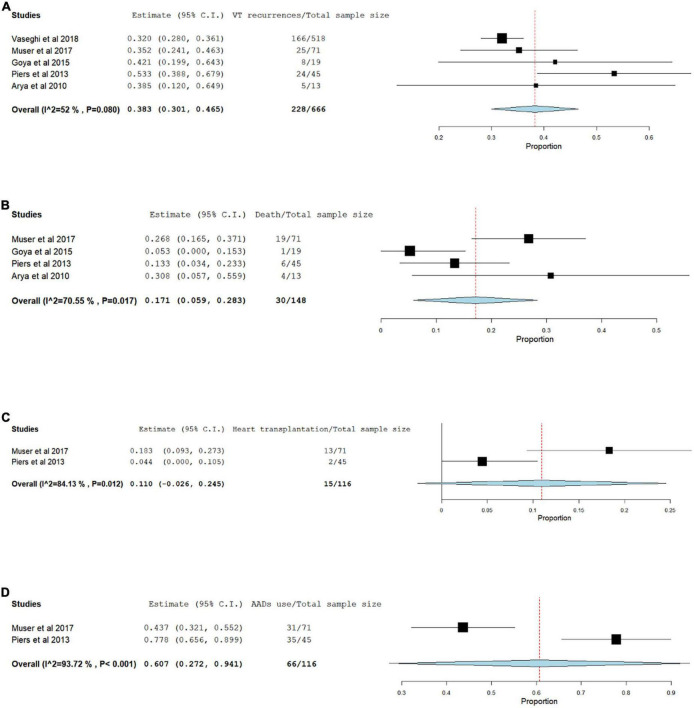
Forest plots for VT recurrence **(A)**, death **(B)**, Heart transplantation **(C)**, AAD use after ablation **(D)**.

During a median follow-up period of 12 to 45 months, the incidence of VT recurrences was 34.2% (95% CI 0.301–0.465, *p* < 0.080) (Figure 3A) and death occurred in 20.2% (95% CI 0.059–0.283, *p* < 0.017) (Figure 3B). In addition, the incidence of the need for heart transplantation pooled from 2 studies was 12.9% (95% CI −0.026–0.245, *P* < 0.012) (Figure 3C) and the AAD use after ablation was also reported only in 2 studies with an estimate of 56.9% (95% CI 0.272–0.941, *P* < 0.001) (Figure 3D).

## Discussion

The VT CA has been increasingly performed nowadays and current guidelines recommend CA as adjunctive treatment to prevent recurrent ICD therapies for VT that cannot be controlled by AADs (22). However, patients with NIDCM are reported to have less favorable outcomes after CA as compared with ICM patients. The HELP-VT (Heart Centre of Leipzig VT) study, which prospectively compared NIDCM with ICM outcomes, showed a poorer VT-free survival at 1 year for DCM of 40.5 vs. 57.0% for ICM patients (7), possibly driven by fundamental differences in the arrhythmic substrate which have important practical and prognostic implications (23).

In this systematic review of the literature and meta-analysis including five observational studies published between 2010 and 2018 with total population number of 666 patients, the acute complete procedural success rate, defined as complete non-inducibility of any VT was achieved in 66% of the patients and procedural complications occurred in 39 of 647 (5.8%) of the patients with the most common complication pericardial effusion/Tamponade.

The complete non-inducibility of VT with PES has been associated with survival benefit and reduction of the likelihood for all-cause mortality in patients with NIDCM in the study done by Dinov et al. (24). In addition persistent VT inducibility at the end of the VT CA procedure was the only independent predictor of long-term VT recurrence in the study done by Muser et al. (18) and Piers et al reached the same conclusion suggesting that non-inducibility may be an appropriate end point in patients with NIDCM (19). On the other hand, the marked variation in the success rates in between the studies reflects the variance in the success rates in the real-world secondary to the difference in the patients’ clinical characteristics, ablation strategies and ablation end points in addition to the study design, and monitoring variance associated with the observational studies.

In our meta-analysis, epicardial mapping and ablation were commonly performed in patients with NIDCM (61.5% and 37% respectively). While evidence supporting routine endo-epi ablation in ICM is less compelling, the benefit of a combined endocardial-epicardial approach for NIDCM was demonstrated in a non-randomized study conducted by Gökoğlan et al including 93 NIDCM patients with the majority (84%) with inferolateral scars (6). The study showed higher short-term procedural success and significantly lower VT recurrence after a first-line endocardial-epicardial substrate ablation approach than with an approach in which epicardial mapping was only performed in case of persistent VT inducibility after endocardial RFCA or lack of an endocardial substrate favoring this approach as an initial strategy for VT ablation in patients with NIDCM (6).

Regarding the long-term outcomes, VT recurrence rates occurred in 34.2% of the patients during a median follow-up of 12 to 45 months. In addition the AAD including Amiodarone, Sotalol and class I AADs were used after ablation in 56.9% although it was reported only in 2 studies. In addition during an average follow up period of 31 months, death was also reported only in 2 studies with an estimate 20.2% of the patients and the need for transplantation was reported in 2 studies as well with an estimate of 12.9%. There was large heterogeneity in VT recurrence, death, and transplantation rates in between the studies likely because of the variation in VT scar locations, potential confounders as age, NYHA class and LVEF, timing of VT ablation and different VT ablation strategies.

## Limitations

The studies included for review were retrospective, single-center observational non-randomized studies, which are subject to confounding factors and bias. In addition most of the studies represented outcomes after a single VT ablation and a repeated VT ablation might have changed the long-term outcomes. Finally there was also discrepancy in the VT ablation strategies among different centers and lack of reporting of important data including type of ablation catheters used for mapping and ablation, in-hospital death, need for heart transplantation and need for redo ablation procedure while on AAD.

## Conclusion

In this meta-analysis, CA of VT in patients with NIDCM was found to be effective and safe approach achieving acute complete procedural success in more than half of the patients using the currently available catheter mapping and ablation techniques with acceptable relatively low procedural complications.

Epicardial approach is usually needed in VT ablation of patients with NIDCM and should be considered as part of initial strategy especially in presence of ECG and CMR findings suggestive of epicardial substrate.

A multicenter randomized trial, although difficult to undertake, would be ideal to look at the short- and long-term outcomes of VT ablation in NIDCM especially with the advances in mapping and ablation techniques and the predictors of success.

## Data availability statement

The raw data supporting the conclusions of this article will be made available by the authors, without undue reservation.

## Author contributions

AA and MI: planning and guarantor. MS and OR: data extraction. MS and KA: methods and results. AA: discussion. All authors contributed to the article and approved the submitted version.

## Abbreviations

AAD: antiarrhythmic drugs
ARVC: arrhythmogenic right ventricular cardiomyopathy
AV: atrio-ventricular
BB: beta blockers
CA: catheter ablation
CRT-D: cardiac resynchronization therapy–defibrillation
CI: confidence Interval
DM: diabetes mellitus
HTN: hypertension
HCM: hypertrophic cardiomyopathy
ICD: implantable cardioverter defibrillator
ICM: ischemic cardiomyopathy
IDCM: idiopathic dilated cardiomyopathy
LV: left ventricle
LVEF: left ventricular ejection fraction
NICM: non-ischemic cardiomyopathy
NIDCM: non-ischemic dilated cardiomyopathy
NR: not reported
PES: programmed electric stimulation
VT: ventricular tachycardia.
